# A meta-analysis of accuracy and sensitivity of chest CT and RT-PCR in COVID-19 diagnosis

**DOI:** 10.1038/s41598-020-80061-2

**Published:** 2020-12-28

**Authors:** Fatemeh Khatami, Mohammad Saatchi, Seyed Saeed Tamehri Zadeh, Zahra Sadat Aghamir, Alireza Namazi Shabestari, Leonardo Oliveira Reis, Seyed Mohammad Kazem Aghamir

**Affiliations:** 1 Urology Research Center, Tehran University of Medical Sciences, Tehran, Iran; 2 Department of Epidemiology and Biostatistics, School of Public Health, Tehran University of Medical Sciences, Tehran, Iran; 3 Faculty of Dentistry, Tehran University of Medical Sciences, Tehran, Iran; 4 Department of Geriatric Medicine, School of Medicine, Tehran University of Medical Sciences, Tehran, Iran; 5 UroScience and Department of Surgery (Urology), School of Medical Sciences, University of Campinas, Unicamp, and Pontifical Catholic University of Campinas, PUC-Campinas, Campinas, São Paulo Brazil

**Keywords:** Cell biology, Genetics, Health care

## Abstract

Nowadays there is an ongoing acute respiratory outbreak caused by the novel highly contagious coronavirus (COVID-19). The diagnostic protocol is based on quantitative reverse-transcription polymerase chain reaction (RT-PCR) and chests CT scan, with uncertain accuracy. This meta-analysis study determines the diagnostic value of an initial chest CT scan in patients with COVID-19 infection in comparison with RT-PCR. Three main databases; PubMed (MEDLINE), Scopus, and EMBASE were systematically searched for all published literature from January 1st, 2019, to the 21st May 2020 with the keywords "COVID19 virus", "2019 novel coronavirus", "Wuhan coronavirus", "2019-nCoV", "X-Ray Computed Tomography", "Polymerase Chain Reaction", "Reverse Transcriptase PCR", and "PCR Reverse Transcriptase". All relevant case-series, cross-sectional, and cohort studies were selected. Data extraction and analysis were performed using STATA v.14.0SE (College Station, TX, USA) and RevMan 5. Among 1022 articles, 60 studies were eligible for totalizing 5744 patients. The overall sensitivity, specificity, positive predictive value, and negative predictive value of chest CT scan compared to RT-PCR were 87% (95% CI 85–90%), 46% (95% CI 29–63%), 69% (95% CI 56–72%), and 89% (95% CI 82–96%), respectively. It is important to rely on the repeated RT-PCR three times to give 99% accuracy, especially in negative samples. Regarding the overall diagnostic sensitivity of 87% for chest CT, the RT-PCR testing is essential and should be repeated to escape misdiagnosis.

## Introduction

In late December of 2019, a cluster of patients was diagnosed with a strange viral pneumonia in Wuhan City, Hubei Province, China, which later was confirmed to be caused by the novel coronavirus (the disease named COVID-19)^1^. Up to now, millions of cases have been identified, causing thousands of deaths at an alarming pace worldwide. Officially, the World Health Organization has declared the pandemic of COVID-19^2^ and due to the non-existence of effective antiviral drug or vaccine, both detecting patient at an early stage and immediate patient isolation play a mandatory role in the fighting against COVID-19^3^.

The chest computed tomography (CT) scan plays a central role on the disease staging and checking the treatment efficacy, while the reverse transcription-polymerase chain reaction (RT-PCR) remains the mainstay of COVID-19 diagnosis^4,5^, though limited to identify the virus, which poses important restrictions^6^.

Recent studies claim that initial chest CT may enable the detection of the disease with higher sensitivity in comparison to RT-PCR^7^. This systematic review and meta-analysis were performed to determine the diagnostic accuracy of the initial chest CT scan compared to RT-PCR in COVID-19 patients.

## Materials and methods

All stages of this study followed the PRISMA guidelines and all relevant English, Chinese, and other language case-series, cross-sectional, and cohort studies were selected and checked for scientific validity.

*Inclusion criteria:* observational epidemiological study design, clear report of the number of positive cases by PCR and chest CT, and the ability to calculate accuracy indicators.

*Exclusion criteria:* case reports or not meeting one or more inclusion criteria.

### Search strategy

All relevant literature from three main databases: MEDLINE (PubMed), Scopus, and EMBASE were explored from January 1st, 2019, to the 21th May 2020, using the keywords “COVID19 virus”, “2019 novel coronavirus”, “Wuhan coronavirus”, “2019-nCoV”, “X-Ray Computed Tomography”, “Polymerase Chain Reaction”, “Reverse Transcriptase PCR”, and “PCR Reverse Transcriptase” (Supplementary file 1). The references of the selected articles were also reviewed.

The variables extracted included the first author name, publication year, country and city of the study, subjects average age, gender, study design, total sample size, true positives, true negatives, false positives, and false negatives.

### Data extraction and statistical analysis

Two researchers (SZA and SSTZ) screened articles separately by checking titles and abstracts. Disagreements were solved by a third one (FKH). Included articles had data of confirmed COVID-19 patients by chest CT scan and quantitative real-time polymerase chain reaction (RT-PCR) and were accessed in full text. The quality assessment was performed by the Newcastle–Ottawa Scale (NOS) assessment tool. The papers that receive scores more than 6 were reflected as the “high quality” and underwent additional meta-analysis steps.

The outcomes of interest, including the CT-scan to identify COVID-19 were submitted to summary receiver operating characteristic (SROC) curve by the random effect model for sensitivity and specificity.

Sensitivity, indicating the capacity of index test to identify patients, considered by “Sensitivity = TP/(TP + FN)”. Specificity as the examination to remove disease-free, calculated by “Specificity = TN/(FP + TN)”. The Metaprop command to calculate sensitivity and specificity excluded studies that have reported 100% sensitivity or specificity.

Positive Predictive Value (PPV) is the probability of disease if the test is positive calculated by "Positive predictive Value = TP/(FP + TP)". Negative Predictive Value (NPV) is the probability of disease-free if the test is negative calculated by "negative predictive value = TN/(FN + TN)".

The Cochran's Q-test of heterogeneity at 5% was used to evaluate statistical heterogeneity based on the Higgins classification in which an I^2^ > 75% means significant heterogeneity.

Deeks’ funnel plot was used to evaluate publication bias by the “Metafunnel”. Briefly, to create the funnel plot, the odds ratio were first calculated using the equations of (TP/FN)/(FP/TN) and after estimating the odds ratio logarithm, the standard error of odds ratio was calculated. Extracted data were collected in Excel 2007 (Microsoft Corporation, Redmond, CA) and analysis was done by using STATA v.14.0SE (College Station, TX, USA) and RevMan 5.

## Results

**Figure 1:**
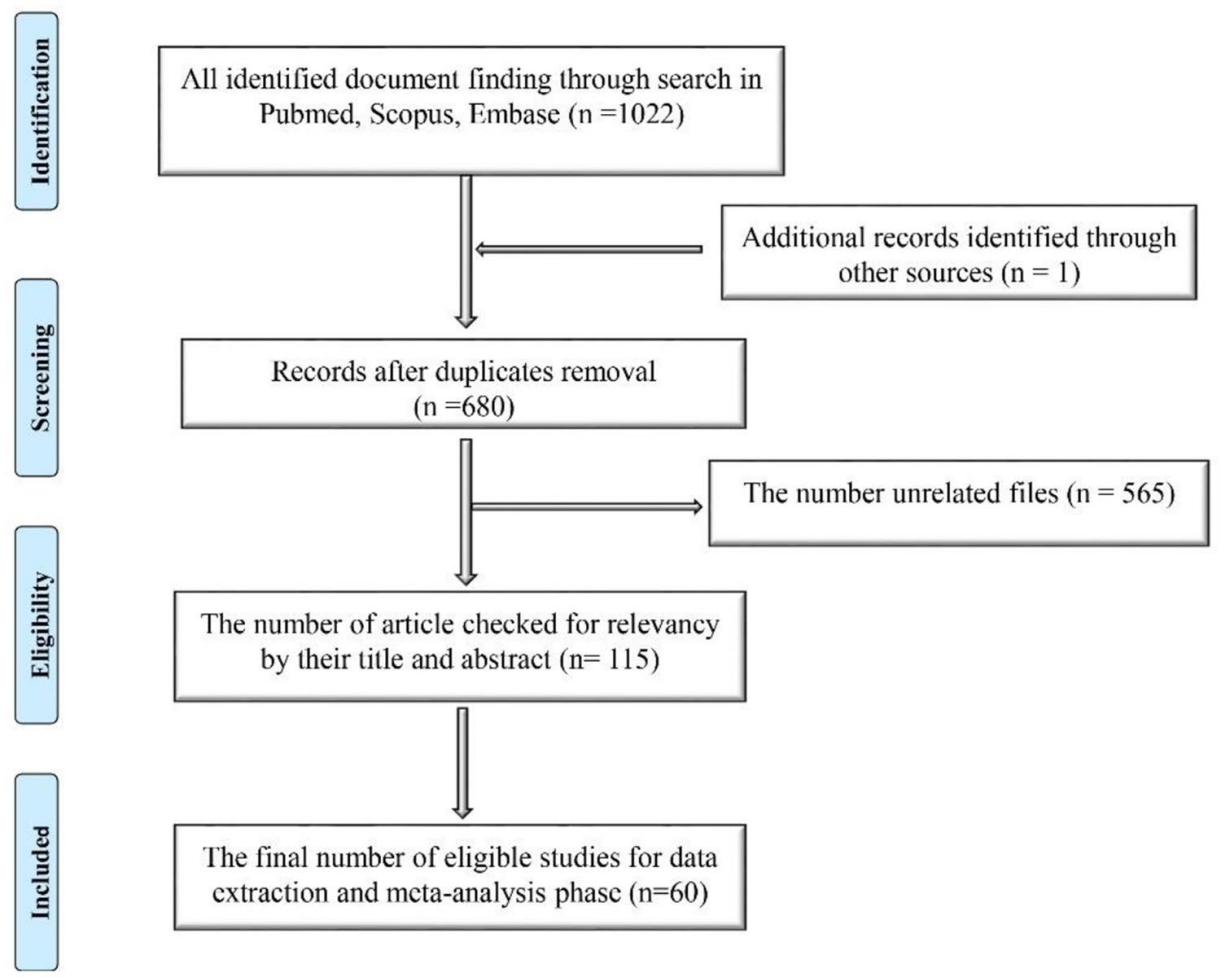
The number of articles during several steps based on the PRISMA flow diagram (2009). (http://www.prisma-statement.org/PRISMAStatement/CitingAndUsingPRISMA.aspx).

Among 1022 identified articles, 115 were considered eligible and after the NOS screening, 60 articles, including 5744 subjects were included, all published in the first quarter of 2020 (Fig. 1).

**Figure 2:**
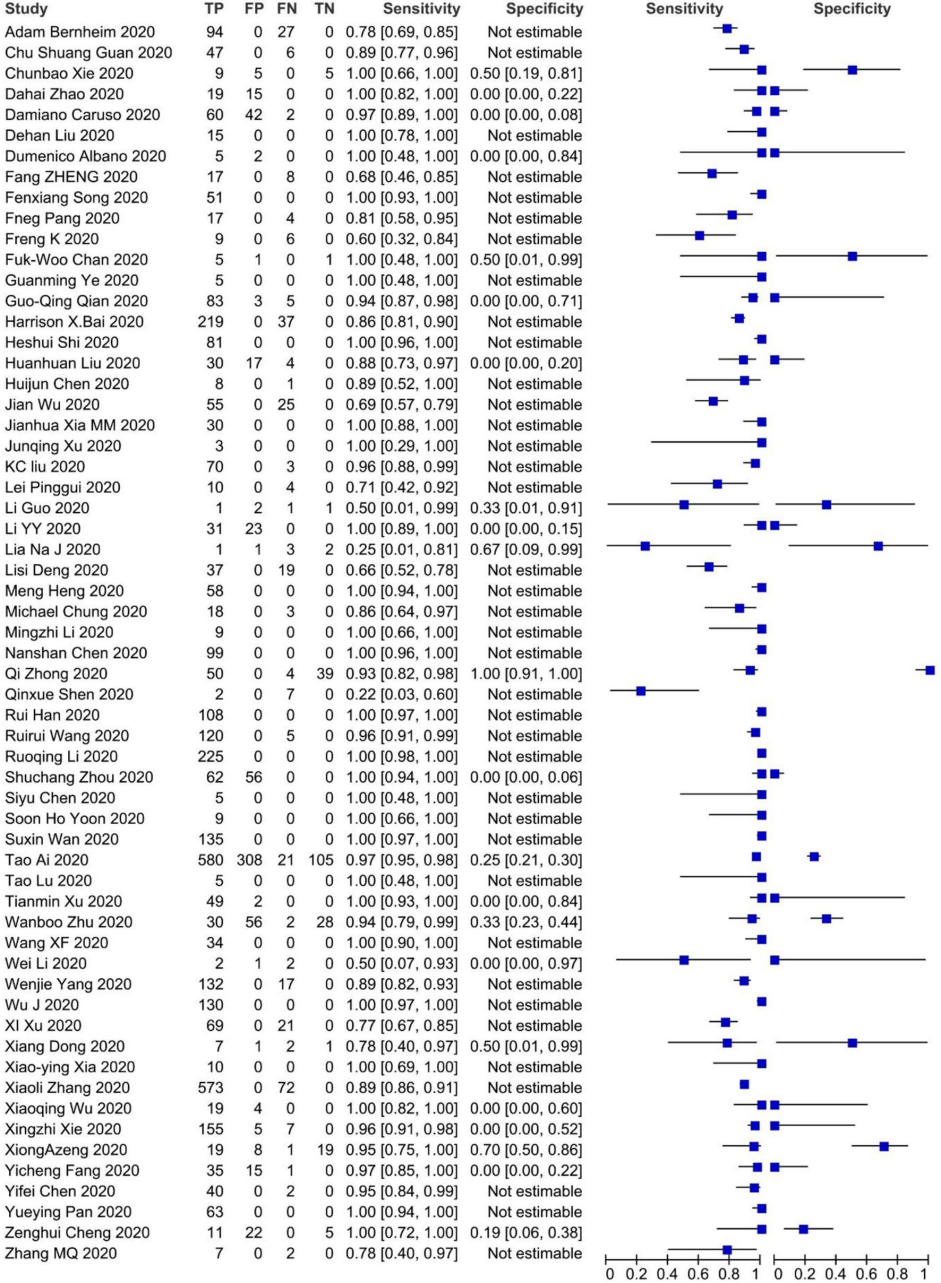
Sensitivity and specificity of 60 included studies.

A summary of the information of included articles is shown in Table 1. The forest plot, False Positives (FP), False Negatives (FN), True Positives (TP), True Negatives (TN), Sensitivity, Specificity, and 95% Confidence Intervals (CI) of each study are shown in Fig. 2 .

**Table 1:** Data of 60 included studies in the data extraction step.

N.	1st author	Country (city)	Gender% male	Age (mean, range, year)	Type of study	Sample size
1	Adam Bernheim^1^	United States (New York)	50%	45, 18–80	Case-series	121
2	Chun Shuang Guan^8^	China (Beijing)	47%	42, 1–86	Case-series	53
3	Chunbao Xie^8^	China (Chengdu)	58%	33, 8–62	Cross-sectional	19
4	Dahai Zhao^9^	China (Anhui)	50%	42, 27–56	Cohort	34
5	Damiano Caruso^10^	Italy (Rome)	53%	57, 18–89	Case-series	158
6	Dehan Liu^11^	China (Wuhan)	0%	32, 23–40	Case-series	15
7	Domenico Albano^12^	Italy (Brescia)	29%	65, 55–79	Case-series	7
8	Fang Zheng^11^	China (Wuhan)	56%	3, 2–9	Case-series	25
9	Fengxiang Song^13^	China (Shanghai)	49%	49, 33–65	Case-series	51
10	Fneg Pang^14^	China (Wuhan)	29%	40, 25–63	Case-series	21
11	Feng Kai^15^	China (Shenzhen)	33%	8, 4–14	Case-series	15
12	Jasper Fuk-Woo Chan^16^	China (Hong Kong)	50%	46, 33–66	Case-series	6
13	Guangming Ye^17^	China (Wuhan)	40%	32, 27–42	Case-series	5
14	Guo-Qing Qian^18^	China (Ningbo)	41%	50, 5–96	Case-series	91
15	Harrison X. Bai^19^	China (Changsha)	NS	NS	Cohort	256
16	Heshui Shi^18^	China (Wuhan)	52%	49.5, 39–61	Case-series	81
17	Huanhuan Liu^20^	China (Shanghai)	14%	20 (2 month–58 years)	Case-series	51
18	Huijun Chen^21^	China (Wuhan)	0%	30, 26–40	Case-series	9
19	Jian Wu^22^	China (Yuncheng)	49%	46, 4–> 65	Case-series	80
20	Jianhua Xia MM^23^	China (Zhejiang)	70%	54.5, 13–74	Cross-sectional	30
21	Junqing Xu^24^	China (Shenzhen)	0%	52, 45–65	Case-series	3
22	KC Liu^22^	China (Hefei)	51%	42, 5–86	Cohort	73
23	Pinggui Lei^25^	China (Guiyang)	57%	47 (12–83)	Case-series	14
24	Li Guo^23^	China (Beijing)	50%	35, 2–64	Cross-sectional	6
25	Li Yuanyuan^26^	China (Wuhan)	46%	52, 25–82	Cross-sectional	54
26	Lia Na Ji^27^	China (Beijing)	NS	NS	Case-series	7
27	Lisi Deng^28^	china (Zhuha)	NS	≥ 18 year	Cohort	56
28	Heng Meng^26^	China (Wuhan)	45%	43	Case-series	58
29	Michael Chung^29^	United States (New York)	61%	51, 29–77	Case-series	21
30	Mingzhi Li^30^	China (Nanchang)	55.5%	43, 31–68	Case-series	9
31	Nanshan Chen^31^	China (Wuhan)	68%	55.5, 21–82	Case-series	99
32	Qi Zhong^32^	China (Wuhan)	23%	32, 28–35	Cohort	93
33	Qinxue Shen^33^	China (Hunan)	33%	8, 1–12	Case-series	9
34	Rui Han^34^	China (Wuhan)	35%	45, 21–95	Case-series	108
35	Ruirui Wang^33^	China (Anhui)	57%	39, 1–80	Case-series	125
36	Ruoqing Li^35^	China (Chongqing)	53%	50	Case-series	225
37	Shuchang Zhou^34^	China (Wuhan)	63%	53, 30–77	Case-series	118
38	Siyu Chen^36^	China (Chongqing)	0%	29, 25–31	Case-series	5
39	Soon Ho Yoon^37^	Korea (Seoul)	44%	54	Case-series	9
40	Suxin Wan^38^	China (Chongqing)	53%	47, 36–55	Cross-sectional	135
41	Tao Ai^36^	China (Wuhan)	46%	51, 2–95	Cross-sectional	1014
42	Tao Lu^32^	China (Sichuan)	20%	52, 41–62	Case-series	5
43	Tianmin Xu^39^	China (Changzhou)	49%	42, 24–65	Cohort	51
44	Wanbo Zhu^34^	China (Hefei)	48%	40, 27–53	Case-series	116
45	Wang XF^33^	China (Shenzhen)	41%	9	Case-series	34
46	Wei Li^18^	China (Zhuhai)	80%	3 (10 month–6 years)	Case-series	5
47	Wenjie Yang^38^	China (Shanghai)	54%	45	Case-series	149
48	Wu Jing^40^	China (Nanjing)	40%	52, 25–80	Case-series	130
49	Xi Xu^41^	China (Guangzhou)	43%	50, 18–86	Case-series	90
50	Xiang Dong^31^	China (Wuhan)	45%	37(2–69)	Case-series	11
51	Xiao-ying Xia^42^	China (Chongqing)	60%	56.5, 43–71	Case-series	10
52	Xiaoli Zhang^43^	China (Zhejiang)	51%	46	Cross-sectional	645
53	Xiaoqing Wu^43^	China (Wuhan)	0%	29, 21–36	Case-series	23
54	Xingzhi Xie^44^	China (Changsha)	NS	NS	Cross-sectional	167
55	Xiong Zeng^40^	China (Changsha)	NS	NS	Cross-sectional	47
56	Yicheng Fang^44^	China (Shanghai)	57%	45, 39–55	Case-series	51
57	Yifei Chen^45^	China (Wuhan)	36%	51 (42–62)	Case-series	42
58	Yueying Pan^46^	China (Wuhan)	52%	45	Case-series	63
59	Zenghui Cheng^46^	China (Shanghai)	NS	NS	Cross-sectional	38
60	Zhang MQ^47^	China (Beijing)	56%	36, 15–49	Case-series	9

*NS* not stated.

Sensitivity ranged from 25 to 100% and the specificity was estimated to vary from 19 to 70%.

**Figure 3:**
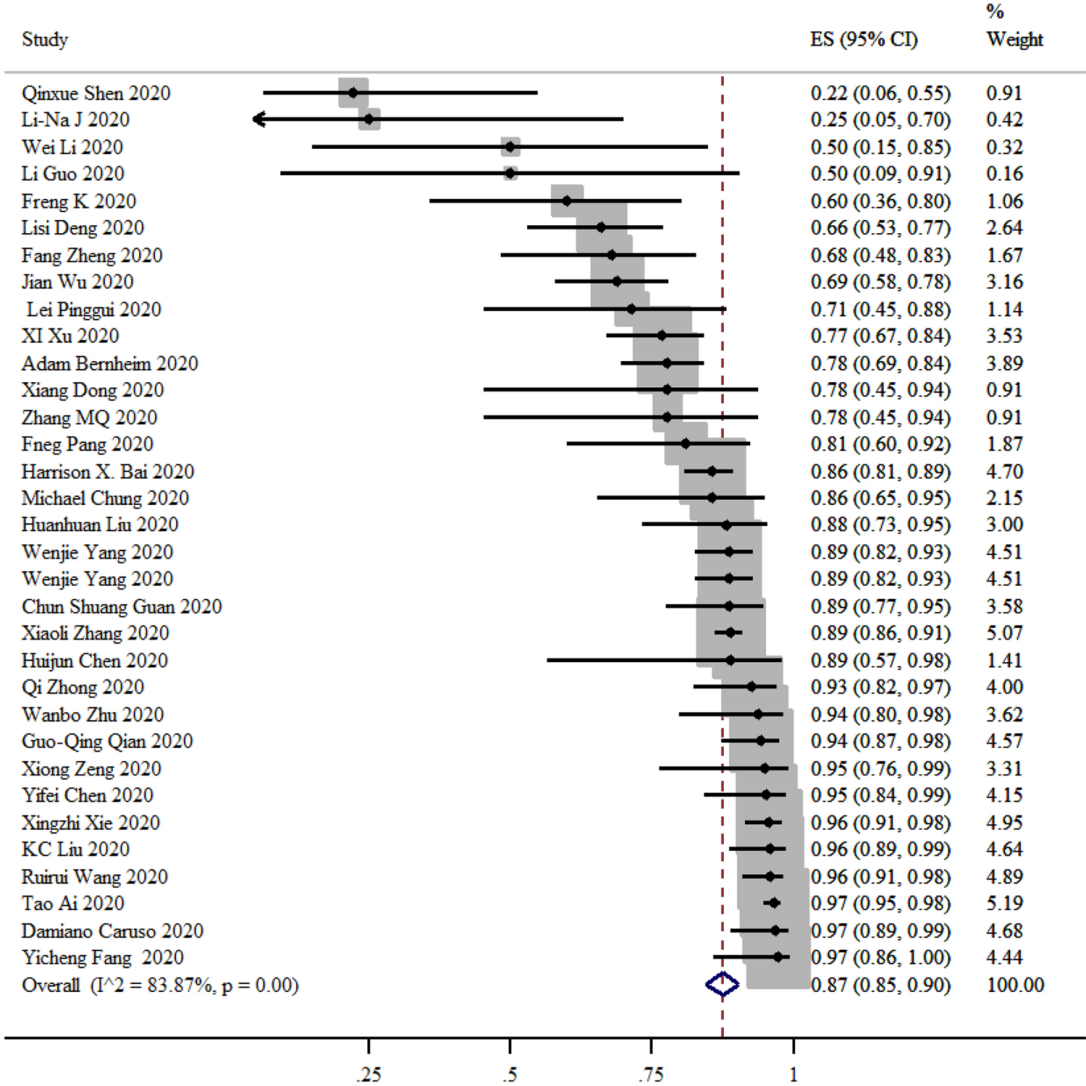
Summary of sensitivity and 95% CI, generated by the STATA.

**Figure 4:**
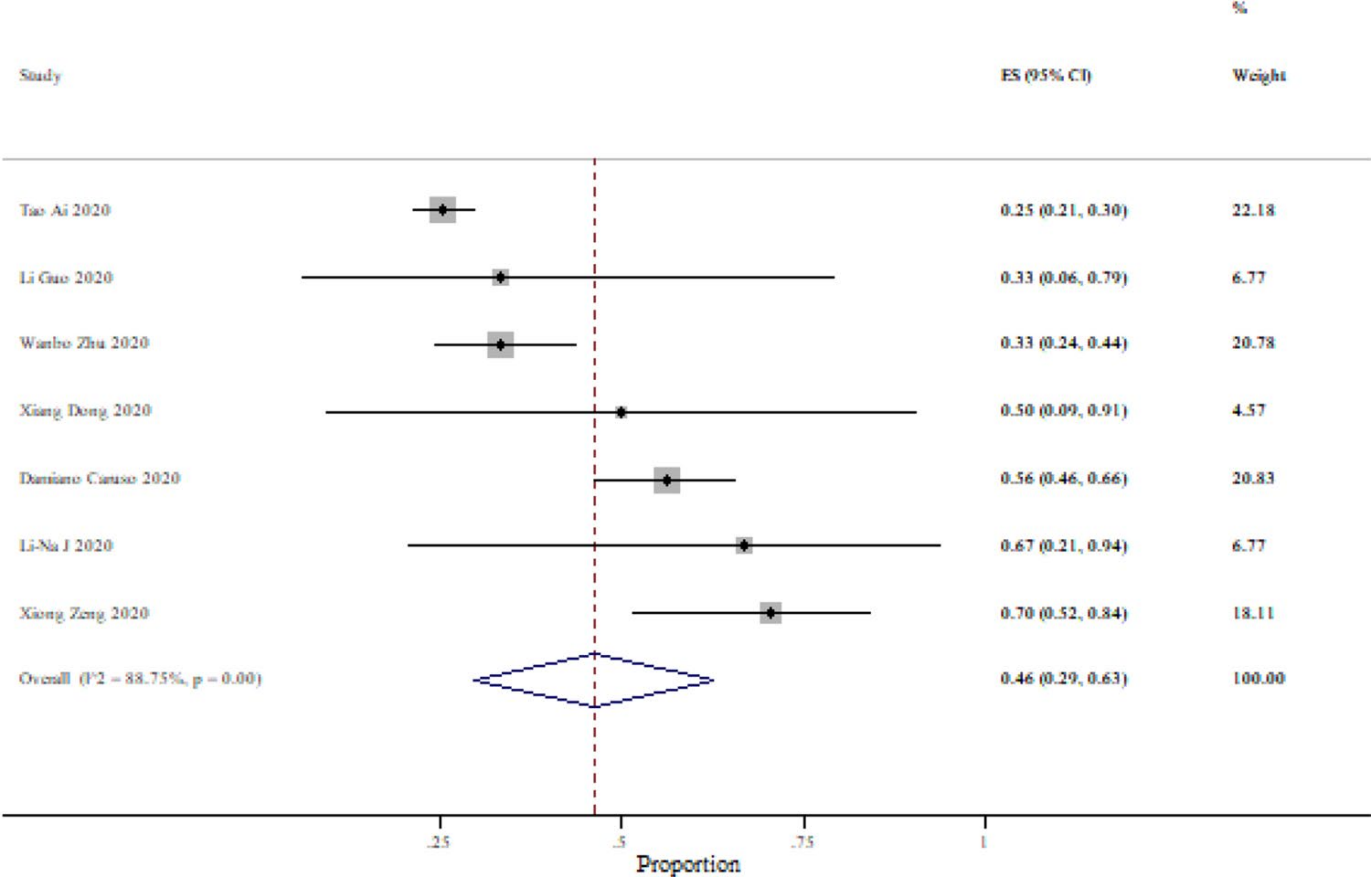
Summary of specificity and 95% CI, generated by the STATA.

After excluding studies reporting 100% sensitivity or specificity, the sensitivity was ranging from 25 to 97% and specificity from 25 to 70% (Figs. 3, 4). Based on 37 studies, the sensitivity of CT compared to PCR was 87% (95% CI 85–90%) and based on seven studies the specificity of CT was 46% (95% CI 29–63%).

**Figure 5:**
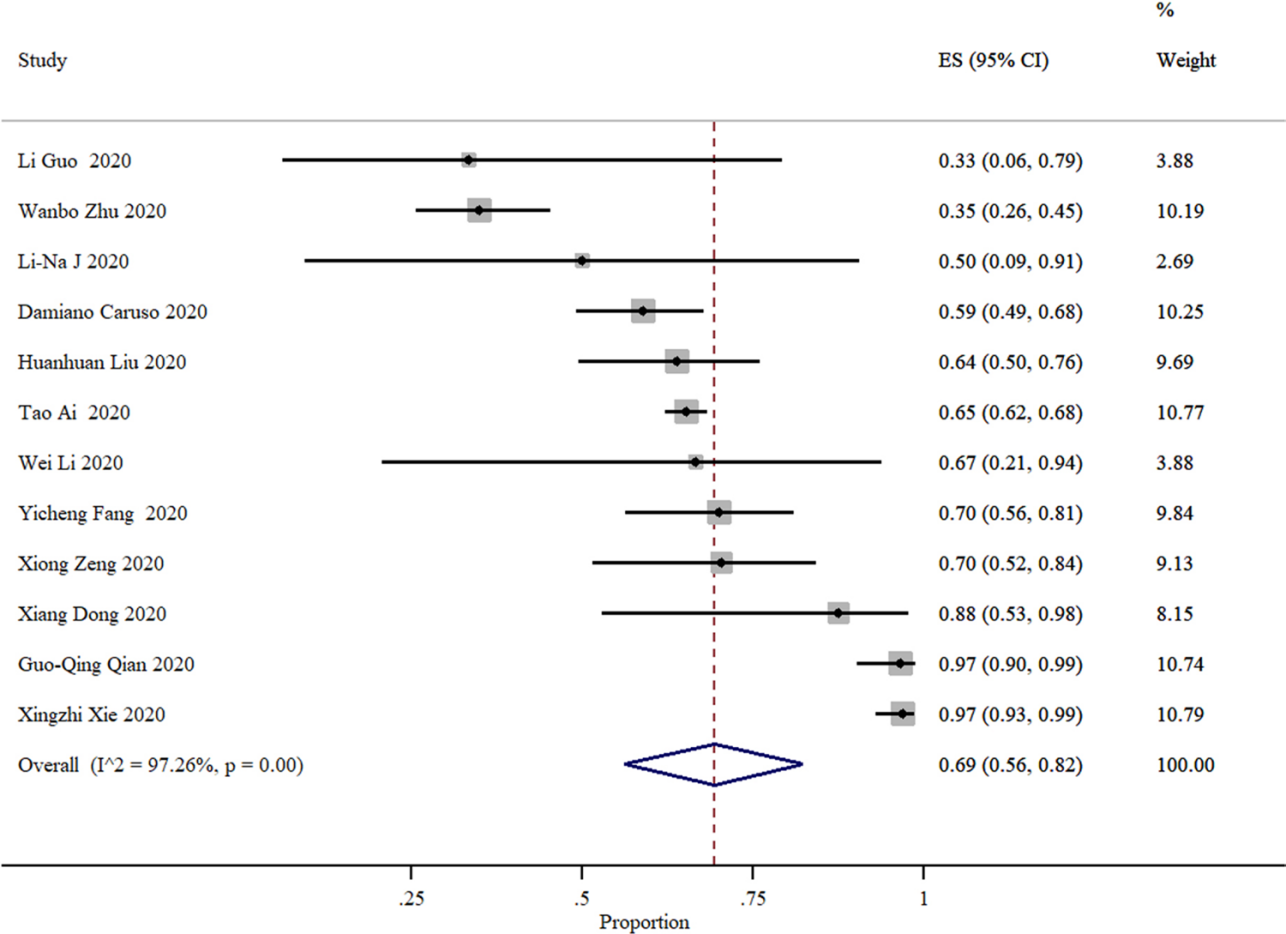
Summary of positive predictive value and 95% CI of 18 studies, generated by the STATA.

**Figure 6:**
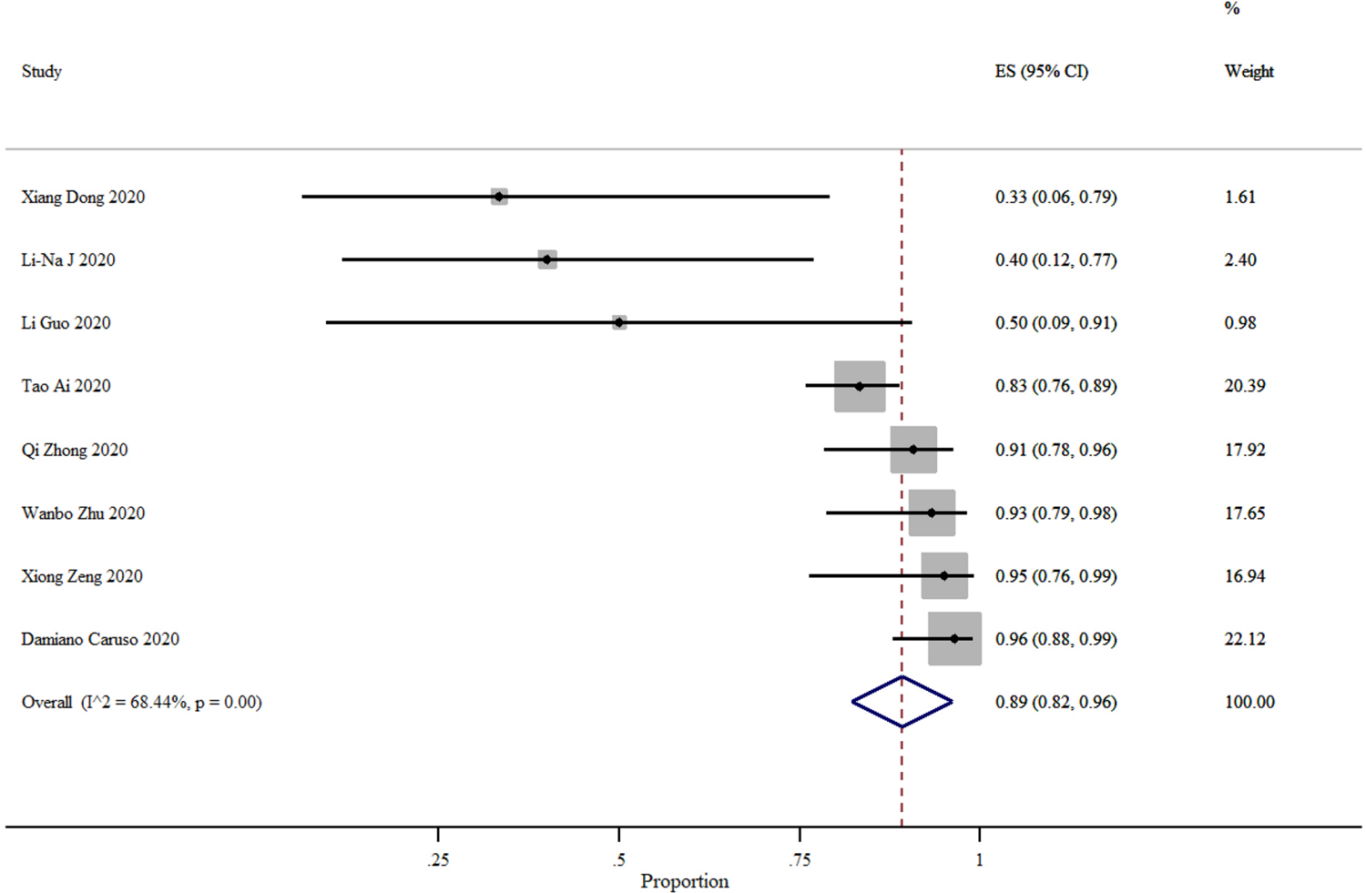
Summary of negative predictive value and 95% CI of six studies, generated by the STATA.

The positive predictive value of CT was 69% (95% CI 56–72%) and the negative predictive value was 89% (95% CI 82–96%) and the variation of the estimated numbers were 33% to 97% and 33% to 96% for PPV and NPV, respectively (Figs. 5, 6).

**Figure 7:**
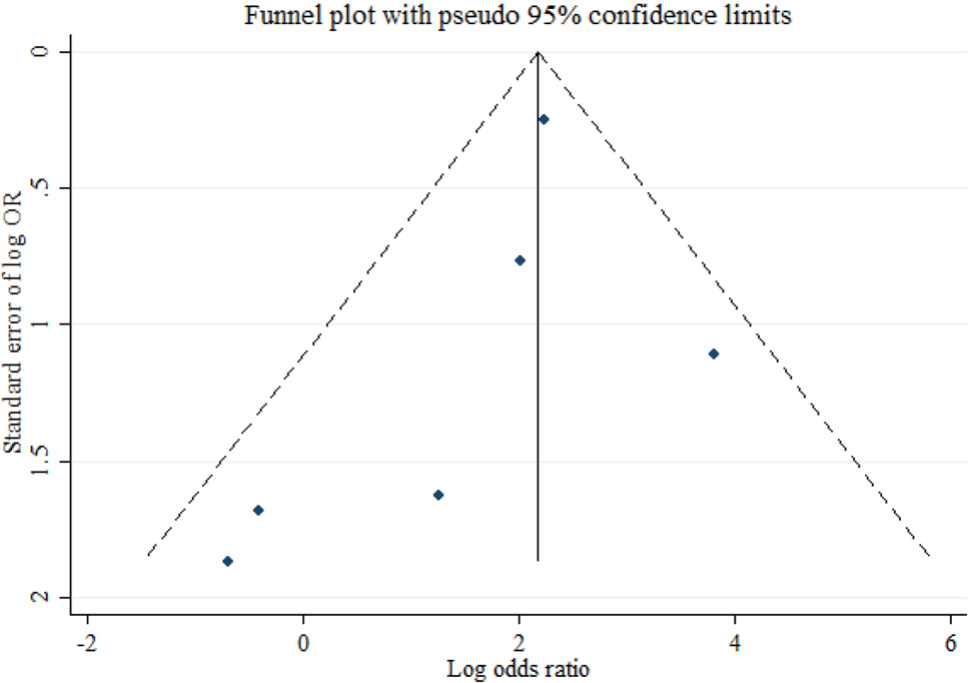
The Deeks’ funnel plot curve for assessment of publication bias.

The symmetry between the two sides of the funnel plot regression line indicates that the included publications are not biased. However, due to a large number of zeros in the FP and TN cells, it was possible to calculate the odds ratio for six studies only, and the interpretation of this plot in our study should be done with caution (Fig. 7).

Table 2 includes 35 studies with the first RT-PCR test of the suspected patients available (subsequent RT-PCR data were included if available). Moreover, the swabs should have been taken from sputum, nasopharyngeal, oropharyngeal, nose, or throat and if a combination was used, nasopharyngeal or throat swab was considered as the primary.

**Table 2:** The number of positive test results in RT-PCR testing and the number of confirmed ones.

N.	First author	N. of total cases	Number of total confirmed patients	N. of patients confirmed with the first RT-PCR test (perc.^a^)	N. of patients confirmed with the second RT-PCR test (perc.^b^)	N. of patients confirmed with the third RT-PCR test (perc.^c^)	N. of patients confirmed with the fourth RT-PCR test (perc.^d^)	N. of Patients confirmed later (perc.^e^)
1	Tao Ai^36^	1014	909	343 (37.7%)	205 (22.6%)	45 (5%)	8 (0.9%)	308 (33.9%)
2	Xingzhi Xie^44^	167	167	162 (97%)	2 (1.2%)	2 (1.2%)	0 (0%)	1 (0.6%)
3	Jian Wu^22^	80	80	41 (51.2%)	30 (37.5%)	9 (11.3%)	0 (0%)	0 (0%)
4	Anne Kimball^48^	82	76	23 (30%)	_^f^	_	_	53 (70%)
5	Li Yuanyuan^49^	54	54	31 (57%)	_	_	_	23 (42.6%)
6	Chenyao Lin^50^	52	52	23 (44%)	_	_	_	29 (56%)
7	Yicheng Fang^44^	51	51	36 (70.6%)	12 (23.6%)	2 (7.8%)	1 (2%)	0 (0%)
8	Qi Zhong^32^	49	49	13 (26.5%)	_	_	_	36 (73.5%)
9	Lorenzo Azzi^51^	25	25	23 (92%)	2 (8%)	-	-	0 (0%)
10	Xiaoqing Wu^43^	23	23	19 (83%)	_	_	_	4 (17%)
11	Qing Chen^52^	9	9	9 (100%)	_	_	_	0 (0%)
12	Li-Na Ji^27^	7	5	4 (80%)	_	_	_	1 (20%)
13	Jasper Fuk-Woo Chan^16^	6	5	4 (80%)	_	_	_	1 (20%)
14	YajunYuan^53^	6	6	6 (100%)	_	_	_	0 (0%)
15	Wei Li^18^	5	5	4 (80%)	0 (0%)	0 (0%)	0 (0%)	1 (20%)
16	Zohre Khodamoradi^54^	4	4	4 (100%)	_	_	_	0 (0%)
17	Li Ni^55^	3	3	1 (33.3%)	0 (0%)	0 (0%)	_	2 (66.7%)
18	Junqing Xu ^24^	3	3	0 (0%)	2 (67%)	1 (33%)	_	0 (0%)
19	Yuanzhe Li^41^	2	2	1 (50%)	1 (50%)	_	_	0 (0%)
20	Michal Paret^56^	2	2	2 (100%)	_	_	_	0 (0%)
21	Zhi-Qun Mao^57^	2	2	2 (100%)	_	_	_	0 (0%)
22	Wendong Hao^44^	1	1	0 (0%)	0 (0%)	0 (0%)	1 (100%)	0 (0%)
23	Peikai Huang^40^	1	1	0 (0%)	0 (0%)	1 (100%)	_	0 (0%)
24	Jinrong Qu^58^	1	1	1 (100%)	_	_	_	0 (0%)
25	Xavier Marchand-Senécal^59^	1	1	1 (100%)	_	_	_	0 (0%)
26	Takeshi Arashiro^55^	1	1	1 (100%)	_	_	_	0 (0%)
27	Hao Feng^30^	1	1	0 (0%)	0 (0%)	0 (0%)	0 (0%)	1 (100%)
28	Ryota Hase^60^	1	1	0 (0%)	1 (100%)	_	_	0 (0%)
29	Yosuke Hirotsu^61^	1	1	1 (100%)	_	_	_	0 (0%)
30	E. Kalafat^62^	1	1	0 (0%)	1 (100%)	_	_	0 (0%)
31	Mojtaba Kamali Aghdam^63^	1	1	1 (100%)	_	_	_	0 (0%)
32	Parisa Karami^64^	1	1	1 (100%)	_	_	_	0 (0%)
33	Dasheng Li^7^	1	1	0 (0%)	0 (0%)	1 (100%)	_	0 (0%)
34	Ding-feng Lv^65^	1	1	0 (0%)	0 (0%)	1 (100%)	1 (100%)	1 (100%)
35	Chaisith Sivakorn^66^	1	1	0 (0%)	1 (100%)	_	_	0 (0%)

^a^Number of primarily confirmed patients divided total confirmed patients.

^b^Number of secondary confirmed patients divided total confirmed patients.

^c^Number of thirdly confirmed patients divided total confirmed patients.

^d^Number of fourthly confirmed patients divided total confirmed patients.

^e^Number of patients who were confirmed later divided by total confirmed patients.

^f^This means the test has not been conducted or reported.

The COVID-19 diagnosis was confirmed by positive result of the first, second, third, and fourth RT-PCR tests and also information of patients who had negative results until the fourth test or no more than one test conducted, but considered as confirmed or most likely ill later according to more RT-PCR tests or examining other swabs, clinical manifestations, typical chest CT scan's features or developmental changes in the series of CT scans or a mixture of prior methods.

In the articles with more than 10 total confirmed patients (first 10 articles included), the RT-PCR test could diagnose 58.9% of the COVID-19 infected patients in the first test, and about 41.1% of infected patients could not be recognized in the first place by RT-PCR test. Among these 10 articles, 5 included the information of second tests (number 1, 2, 3, 7 and 9). In these five articles, the mean percentage of secondary diagnosed patients divided by total confirmed patients is 18.6%. Out of 4 articles (number 1, 2, 3 and 7) with the exact data of thirdly and fourthly conducted tests, the mean percentage of positivity are 6.3% and 0.7%, respectively. Moreover, the percentage of patients who were not diagnosed after 4 times of repeating the test is 8.6% (in the previous 4 articles). The numbers and sequences of primers and probes could be influential on PCR sensitivity and specificity which were surveyed in Table 3.

**Table 3:** From the information illustrated in Table 2, those which their primer and probe's data were available.

N.	First author	Total N. of cases	N. of patients confirmed with the first RT-PCR test	N. patients confirmed later	Primarily confirmed patients divided total confirmed patients (%)	Number of sets (primer and probe)	Type of genes
1	Jian Wu^22^	80	41	39	51.2	2	N and ORF1ab
2	Anne Kimball^48^	82	23	53	30	2	N
3	Chenyao Lin^50^	52	23	29	44	2	N and ORF1ab
4	Qing Chen^52^	9	9	0	100	3	RdRP, E and N
5	Jasper Fuk-Woo Chan^16^	6	4	1	80	2	RdRP and S
6	Wendong Hao^44^	1	0	1	0	1	ORF1ab
7	Xavier Marchand-Senécal^59^	1	1	0	100	1	RdRP
8	Yosuke Hirotsu^61^	1	1	0	100	7	N gene
9	Ding-feng Lv^65^	1	0	1	0	1	ORF1ab or N gene

As we can see in Table 3, in the case report by Wendong Hao^44^, using one pair of primer and probe did not indicate a positive result at first but in the fourth repeated test. In another case report by Feng Ly^65^, the oropharyngeal swab by detection of N gene showed a positive result in the third, fourth, and fifth time, whereas ORF1ab detection showed a positive result in the fifth examination. On the other hand, Xavier Marchand-Senécal et al.^67^ and Yosuke Hirotsu et al.^61^ reported 2 cases that were diagnosed initially with one pair of primer and probe of PCR test.

By using 2 pairs of primer and probe, the mean of initially detecting patients divided by total confirmed patients is 51.3% in the 4 studies above. Also, Qing Chen^52^ findings with the utilization of 3 pairs of primer and probe caused 100% of initially discovering COVID-19 patients^52^. Moreover, using 7 sets of primers and probes also resulted positively for the first test^61^. Based on limited data available, it seems that the greater the number of primer and probe, more likely to initially detect patients, although more specific information is needed from future studies.

## Discussion

Considering the outcomes of RT-PCR as a reference, in our meta-analysis, the sensitivity and specificity of initial chest CT scan for detecting patients, who were highly suspicious for COVID-19, were 87% and 43% respectively. The PPV and NPV of CT scans were 67% and 84% respectively.

It means that 67% of individuals with positive chest CT scans had positive RT-PCR and 84% of individuals with negative chest CT scans had negative RT-PCR. So, a chest CT scan may have beneficial diagnostic features as adjuvant diagnostic tool compared to RT-PCR^36,68^.

Tao Ai and colleagues studying 1014 patients, 888 (88%) with a positive chest CT scan and 601 with a positive RT-PCR for COVID-19, described 97%, 25%, 65%, and 83% of sensitivity, specificity, PPV, and NPV for the CT scan, respectively. The relatively high sensitivity and low specificity in this study might be related to the low odds ratio of positive RT-PCR, considered as the reference test^69^, as suggested by the World Health Organization (WHO)^70^.

Some patients have typical chest CT scan findings and symptoms for COVID-19 but their initial RT-PCR results were negative agreeing with previous research reports^19,36,44^. Fang et al. described 15 out of 51 patients who have an initial negative RT-PCR while their chest CT scan was positive^36,44^, so it is very important to pay attention to chest CT scan, epidemiologic features, and clinical symptoms. Furthermore, a combination of humoral (IgG-IgM antibody) and cellular immunity, in addition to RT-PCR could refine the detection of COVID-19^23,49^.

The results of Chan and colleagues indicated that among 273 specimens (15 COVID-19 positives), the RdRp-P2 test showed 77 positive specimens and the RdRp/Hel test showed 42 positives. Moreover, RdRp/Hel analysis did not cross-reacted with any human coronaviruses or other respiratory pathogens while RdRp-P2 analysis reacted to SARS-CoV either^71^. Another study expressed that the sensitivity of N gene assay in finding the positive samples is 10 times higher than the ORF-1b gene assay^72^.

In February, 280 suspected patients with clinical manifestations of COVID-19 were tested in the Marseille hospital. None of the patients were positive for SARS-COV-2^73^. Guo-Qing Qian et al. reported that all the patients, except three of them, were confirmed with the second RT-PCR test^18^. In the study by Tao Ai et al., it is highlighted that out of 1014 patients, 308 patients with negative PCR results, were strongly perceived as infected by clinical manifestations and CT scans. The percentage of a positive test for the first, second, third, and fourth tests were 37.7%, 22.6%, 5%, and 0.9%, respectively. Unfortunately, about 33.9% of the patients could not be diagnosed even with the fourth test. CT scans were positive in 580 of 601 (97%) COVID-19 confirmed patients by the RT-PCR test and in 308 of 413(74.6%) patients with negative RT-PCR assay^74^. In a retrospective analysis, among 51 patients, 98% (50/51) had abnormal CT, besides 70.6% (36/51) had positive PCR assay initially and about 30% of the tests became positive after second, third, and fourth scanning^44^.

A study of five children suggested that four children had positive PCR outcomes within the first assay, but one with COVID-19 suggestive CT findings turned positive after six times of examining^35^. It is also possible that negative RT-PCR with three times of repeating, turns positive on the fourth test, while CT demonstrated typical features such as GGO^44^. Chest CT presented rapidly developing multiple patchy consolidations and GGOs in both lungs of a case reported by J Wei, while in the later stage, there was the development of fibrosis. So with high-resolution CT, it will be easier to find GGOs in the early stage^75^.

What stands out from Table 2 is that 58.9% of the infected COVID-19 patients could be recognized in the first test and about 18.6%, 6.3% and 0.7% could be diagnosed in the further second, third and fourth tests, respectively. Besides, about 9% of the infected patients have not been detected, even after the fourth test. According to the results and due to the PCR's cost and time consumption, it seems that repeating the test up to 3 times is reasonable in patients with initially negative results (with 24 h to 3 days' time interval based on literature). Also, CT scan findings and clinical manifestation should be encountered in all patients, especially in suspected ones with multiple negative PCRs.

About 80% of COVID-19 patients have mild disease and just about 15% of them will reach severe stages. Positive RT-PCR results usually have a high positive predictive value, but negative RT-PCR should be repeated three times to increase the negative predictive value up to 98% (57% at first test, 34% at second one, and 7% at third time).

If the patient's death is due to COVID-19, but their PCR is negative, even if their chest CT is positive, their cause of death would not report COVID-19. Some patients with negative PCR result die, but based on our results, 87% of them are Covid-19 positive and their disease should be confirmed by repeating PCR for up to three times.

On the other hand, the exact place of chest CT is for staging the COVID-19 disease as mild, moderate, and severe, instead of being a screening tool. Some antibody and serology testing can support the RT-PCR test. A study in two patients with COVID-19 pneumonia by Lin and colleagues indicated the presence of ground-glass lesions and patchy consolidations in repeated chest CT^76^. Also, the lesions were classically accompanied by bronchial bundles or subpleural lesions. In patients who have a fever but not having the previous contact with the epidemic area, the appropriate finding of the COVID-19 RNA is compulsory to guarantee the high efficacy of treatment^76^.

We acknowledge that our study had some limitations: (1) the specificity of CT scan was not as reliable as the sensitivity, due to the majority of studies' nature, which were case-series and the number of true negative patients in those studies were zero. (2) It has been postulated in Bernheim et al.'s study that the chance of detecting lung involvement in chest CT scan will be increased if the duration between symptom onset and initial chest CT scan rises and this duration was different among 60 studies.

In conclusion, the results of the present systematic review and meta analysis shed new light on the comparison between chest-CT scan and rRT-PCR validity in terms of diagnosis in patients with COVID-19. Due to lower diagnostic sensitivity of chest-CT scan in comparison to rRT-PCR, performing rRT-PCR is mandatory for any individuals with suspicious symptoms. Nevertheless, the initial negative rRT-PCR result is not fully able to roll out COVID-19 in all cases and because of that, repeating the test for three times is vital to roll out COVID-19.

## Supplementary Information

Supplementary Information 1.

Supplementary Information 2.

## Data Availability

Information, data, and photos will be provided if they are requested.
