# Supplementary material for: A meta-analysis of accuracy and sensitivity of chest CT and RT-PCR in COVID-19 diagnosis
Source: Sci Rep. 2020 Dec 28;10:22402. doi: 10.1038/s41598-020-80061-2 (PMC7769992; doi:10.1038/s41598-020-80061-2)
Supplement: Supplementary file 1 — Supplementary Information 1. [file 41598_2020_80061_MOESM1_ESM.docx]

COVID 19 Search Syntax

**Mesh Terms:**

- Wuhan coronavirus
- Wuhan seafood market pneumonia virus
- COVID19 virus
- COVID-19 virus
- coronavirus disease 2019 virus
- SARS-CoV-2
- SARS2
- 2019-nCoV
- 2019 novel coronavirus

• X-Ray Computed Tomography

• Tomography, X-Ray Computerized

• Tomography, X Ray Computerized

• Computed X Ray Tomography

• X-Ray Computer Assisted Tomography

• X Ray Computer Assisted Tomography

• Tomography, X-Ray Computer Assisted

• Tomography, X Ray Computer Assisted

• Computerized Tomography, X Ray

• Computerized Tomography, X-Ray

- Polymerase Chain Reaction, Reverse Transcriptase
- Reverse Transcriptase PCR
- PCR, Reverse Transcriptase
- Transcriptase PCR, Reverse

***Pubmed***

### History

[Download history](https://www.ncbi.nlm.nih.gov/pubmed?p$l=Email&Mode=download&dlid=history&filename=history.csv&db=pubmed&historyid=NCID_1_63205502_130.14.18.48_5555_1583827572_1774901699_0MetA0_S_HStore&p$debugoutput=off)[Clear history](https://www.ncbi.nlm.nih.gov/pubmed/advanced)

| Recent queries | | | | |
| --- | --- | --- | --- | --- |
| Search | Add to builder | Query | Items found | Time |
| [#7](https://www.ncbi.nlm.nih.gov/pubmed/advanced) | [Add](https://www.ncbi.nlm.nih.gov/pubmed/advanced) | Search **((((((("x ray computed tomography"[Title/Abstract]) OR "Tomography, X-Ray Computerized"[Title/Abstract]) OR "ct scan"[Title/Abstract]) OR "x ray"[Title/Abstract]) OR Computerized Tomography[Title/Abstract]) AND ("2019.01.01"[Date - Publication] : "2020.03.10"[Date - Publication]))) AND ((((((("wuhan coronavirus"[Title/Abstract]) OR "coronavirus e"[Title/Abstract]) OR coronavirus[Title/Abstract]) OR SARS-CoV-2[Title/Abstract]) OR 2019-nCoV[Title/Abstract]) OR novel coronavirus[Title/Abstract]) AND ("2019.01.01"[Date - Publication] : "2020.03.10"[Date - Publication]))** | [27](https://www.ncbi.nlm.nih.gov/pubmed/?cmd=HistorySearch&querykey=7) | 04:28:20 |
| [#6](https://www.ncbi.nlm.nih.gov/pubmed/advanced) | [Add](https://www.ncbi.nlm.nih.gov/pubmed/advanced) | Search **((((("x ray computed tomography"[Title/Abstract]) OR "Tomography, X-Ray Computerized"[Title/Abstract]) OR "ct scan"[Title/Abstract]) OR "x ray"[Title/Abstract]) OR Computerized Tomography[Title/Abstract]) AND ("2019.01.01"[Date - Publication] : "2020.03.10"[Date - Publication])** | [30202](https://www.ncbi.nlm.nih.gov/pubmed/?cmd=HistorySearch&querykey=6) | 04:26:27 |
| [#4](https://www.ncbi.nlm.nih.gov/pubmed/advanced) | [Add](https://www.ncbi.nlm.nih.gov/pubmed/advanced) | Search **(((((((("polymerase chain reactions"[Title/Abstract]) OR "reverse transcriptase pcr"[Title/Abstract]) OR "transcriptase ploymerase chain reaction"[Title/Abstract]) AND ("2019.01.01"[Date - Publication] : "2020.03.10"[Date - Publication]))) OR "pcr"[Title/Abstract]) AND ("2019.01.01"[Date - Publication] : "2020.03.10"[Date - Publication]))) AND ((((((("wuhan coronavirus"[Title/Abstract]) OR "coronavirus e"[Title/Abstract]) OR coronavirus[Title/Abstract]) OR SARS-CoV-2[Title/Abstract]) OR 2019-nCoV[Title/Abstract]) OR novel coronavirus[Title/Abstract]) AND ("2019.01.01"[Date - Publication] : "2020.03.10"[Date - Publication]))** | [157](https://www.ncbi.nlm.nih.gov/pubmed/?cmd=HistorySearch&querykey=4) | 04:18:59 |
| [#3](https://www.ncbi.nlm.nih.gov/pubmed/advanced) | [Add](https://www.ncbi.nlm.nih.gov/pubmed/advanced) | Search **(((((("polymerase chain reactions"[Title/Abstract]) OR "reverse transcriptase pcr"[Title/Abstract]) OR "transcriptase ploymerase chain reaction"[Title/Abstract]) AND ("2019.01.01"[Date - Publication] : "2020.03.10"[Date - Publication]))) OR "pcr"[Title/Abstract]) AND ("2019.01.01"[Date - Publication] : "2020.03.10"[Date - Publication])** | [38038](https://www.ncbi.nlm.nih.gov/pubmed/?cmd=HistorySearch&querykey=3) | 04:15:29 |
| [#2](https://www.ncbi.nlm.nih.gov/pubmed/advanced) | [Add](https://www.ncbi.nlm.nih.gov/pubmed/advanced) | Search **((("polymerase chain reactions"[Title/Abstract]) OR "reverse transcriptase pcr"[Title/Abstract]) OR "transcriptase ploymerase chain reaction"[Title/Abstract]) AND ("2019.01.01"[Date - Publication] : "2020.03.10"[Date - Publication])** | [331](https://www.ncbi.nlm.nih.gov/pubmed/?cmd=HistorySearch&querykey=2) | 04:14:11 |
| [#1](https://www.ncbi.nlm.nih.gov/pubmed/advanced) | [Add](https://www.ncbi.nlm.nih.gov/pubmed/advanced) | Search **(((((("wuhan coronavirus"[Title/Abstract]) OR "coronavirus e"[Title/Abstract]) OR coronavirus[Title/Abstract]) OR SARS-CoV-2[Title/Abstract]) OR 2019-nCoV[Title/Abstract]) OR novel coronavirus[Title/Abstract]) AND ("2019.01.01"[Date - Publication] : "2020.03.10"[Date - Publication])** | [1377](https://www.ncbi.nlm.nih.gov/pubmed/?cmd=HistorySearch&querykey=1) | 04:11:24 |


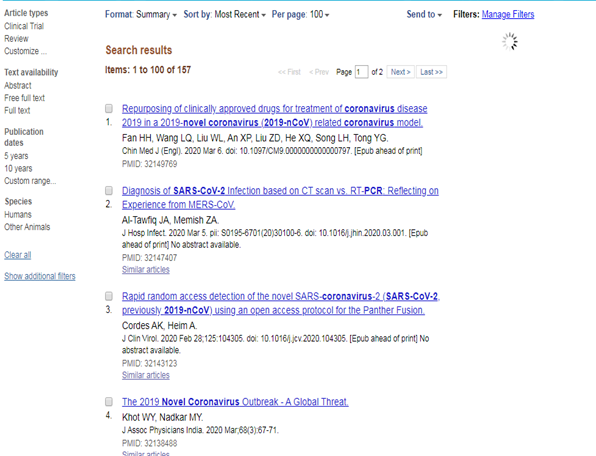


***Scopus***

***
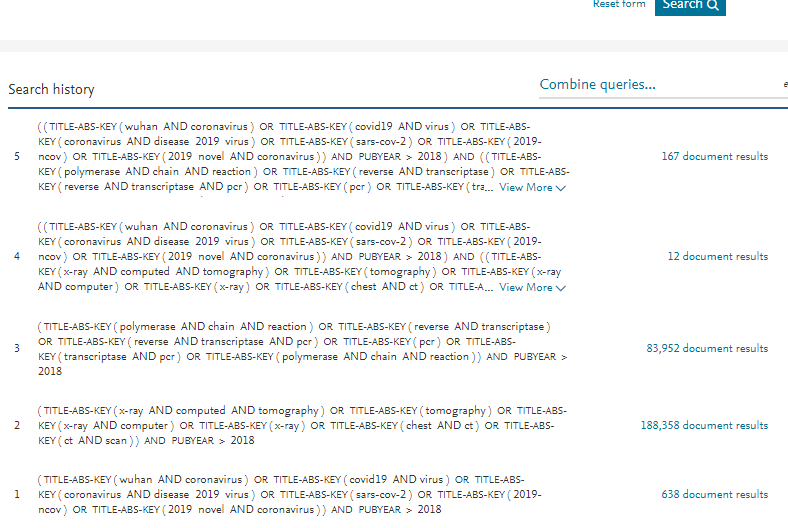
***

***
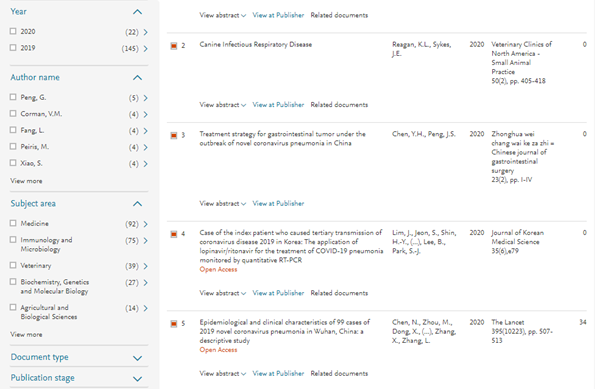
***

***Embase***


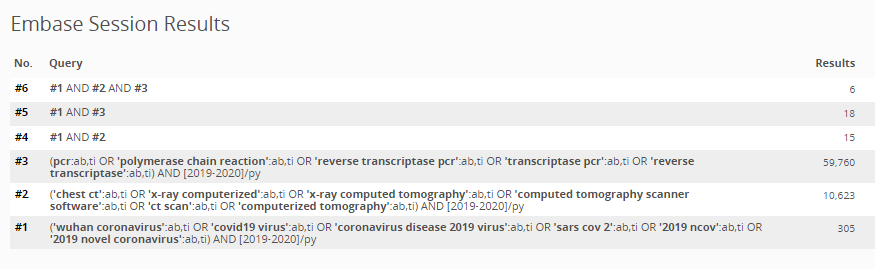


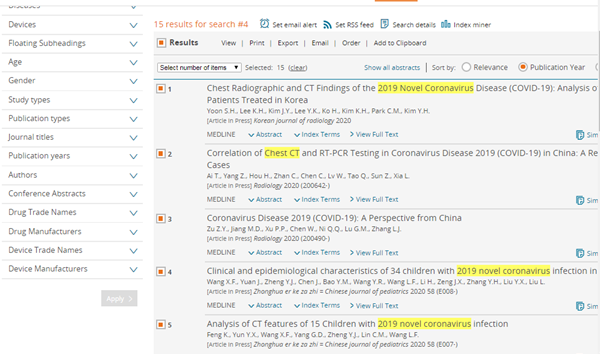


***Cochrane***

***
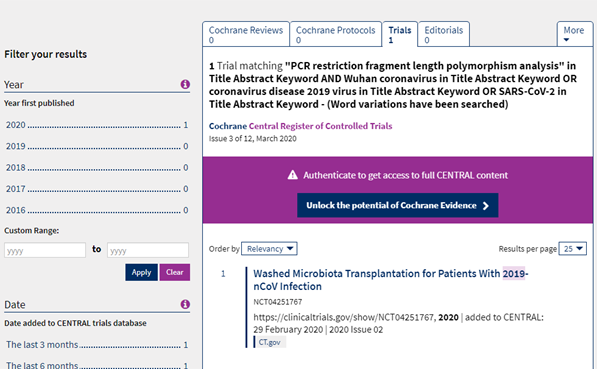
***

***
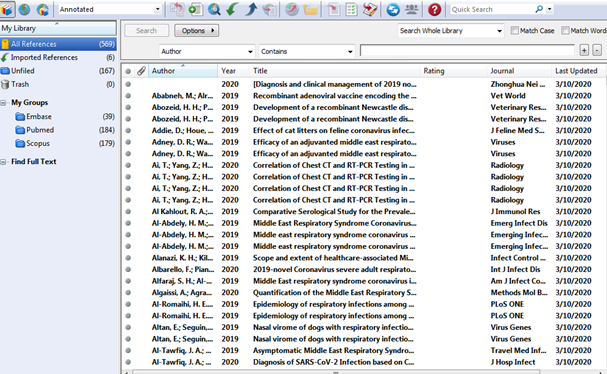
***

***
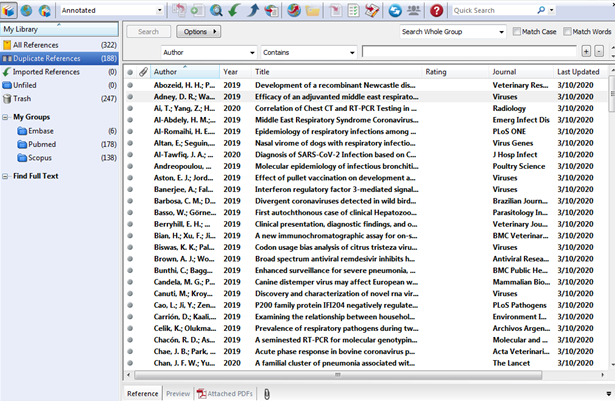
***
